# Lipid-Mediated Assembly of Biomolecular Condensates: Mechanisms, Regulation, and Therapeutic Implications

**DOI:** 10.3390/biology14091232

**Published:** 2025-09-10

**Authors:** Shijie Ma, Zheng Yang, Chang Du, Binjie Gan, Tong Tang

**Affiliations:** 1 Crop Research Institute, Anhui Academy of Agricultural Sciences, Hefei 230031, China; mashijieaaas@163.com; 2 Zhengzhou Research Base, State Key Laboratory of Cotton Bio-breeding and Integrated Utilization, School of Agricultural Sciences, Zhengzhou University, Zhengzhou 450001, China; yangz@zzu.edu.cn; 3 Guangdong Provincial Key Laboratory of Biotechnology for Plant Development, School of Life Science, South China Normal University, Guangzhou 510631, China; duchang@m.scnu.edu.cn; 4 Department of Computer Science and Information Technologies, Elviña Campus, University of A Coruña, 15001 A Coruña, Spain

**Keywords:** biomolecular condensates, liquid–liquid phase separation, lipid rafts, membrane organization, phase coupling, membrane anchoring, therapeutic modulation

## Abstract

Cellular organization relies on both membrane-bound organelles and membraneless biomolecular condensates formed through liquid–liquid phase separation. Recent discoveries reveal intricate coupling between lipid membrane organization and condensate assembly, reshaping our understanding of cellular compartmentalization. This review synthesizes multidisciplinary research using advanced techniques including super-resolution microscopy, fluorescence recovery after photobleaching, and in vitro reconstitution to examine lipid-condensate interactions. Lipid membranes serve as nucleation platforms that reduce critical concentrations for condensate formation by orders of magnitude through membrane anchoring and thermodynamic coupling, creating specialized microenvironments that substantially enhance enzymatic activities. Key regulatory mechanisms include phosphorylation-driven assembly and disassembly, membrane composition effects from cholesterol content and fatty acid saturation, and environmental factors such as calcium and pH. These interactions drive signal transduction through receptor clustering, membrane trafficking via organized domains, and stress responses through protective condensate formation. Dysregulation of lipid-condensate coupling, including aberrant phase transitions and membrane dysfunction, underlies metabolic disorders and neurodegenerative diseases. This coupling represents a fundamental organizing principle with significant therapeutic potential. Current challenges include developing quantitative methods for characterizing condensate dynamics in complex cellular environments and translating molecular mechanisms into clinical applications. Future progress requires interdisciplinary approaches combining advanced experimental techniques, computational modeling, and standardized protocols to advance both fundamental understanding and therapeutic innovations.

## Simple Summary

Cells function like miniature factories, requiring precise organization to operate effectively. Traditionally, this was thought to depend solely on compartments enclosed by fatty barriers known as membranes. Recent findings reveal that cells also form wall-less, droplet-like clusters from proteins and other molecules, similar to how oil separates in water. This review examines the interactions between these droplets and membranes, addressing how cells manage intricate activities. The objective was to compile insights from diverse studies on how membranes facilitate droplet formation, the factors regulating them, such as protein modifications or environmental shifts, and their involvement in tasks like signal transmission, material transport, and stress adaptation. Results indicate that these interactions are finely tuned and essential for normal cell operations, with disruptions linked to conditions like metabolic disorders. In conclusion, this droplet-membrane partnership unveils a core mechanism for cellular order and flexibility, offering potential for innovative therapies that target these processes to enhance societal health by tackling diseases at their fundamental level.

## 1. Introduction

Cellular compartmentalization is essential for the coordination of biochemical reactions, signal transduction, and adaptive responses to environmental stimuli. Traditionally, this compartmentalization has been primarily attributed to membrane-bound organelles, such as mitochondria, the endoplasmic reticulum, and lysosomes, which isolate specific molecular processes through lipid bilayers [1]. However, research over the past decade has revealed an emerging and widely existing organizational mechanism: membrane less biomolecular condensates formed through liquid–liquid phase separation (LLPS) [2,3]. These dynamic structures include nucleoli, stress granules, P-bodies, and Cajal bodies, which can selectively enrich proteins, nucleic acids, and other biomolecules in the absence of membrane boundaries, thereby promoting rapid and reversible cellular responses [4,5]. Concurrently, cellular membranes organize functionally through lipid phase separation, generating cholesterol- and sphingolipid-enriched liquid-ordered (Lo) domains known as lipid rafts. These specialized membrane regions facilitate immune signaling, vesicle trafficking, and protein organization through their characteristic features: enhanced lipid chain order, reduced molecular fluidity, and selective enrichment of specific membrane components [6].

The formation of biomolecular condensates relies on multivalent interactions involving intrinsically disordered regions (IDRs), low-complexity domains (LCDs) in proteins, and nucleic acids (particularly RNA) [7]. These interactions involve various molecular forces, including π-π stacking, electrostatic interactions, hydrophobic effects, and cation-π interactions, which collectively drive the phase separation process [8]. These forces contribute differentially to condensate stability: nucleoli maintain high stability through strong RNA-protein networks, stress granules exhibit dynamic assembly regulated by phosphorylation, P-bodies demonstrate intermediate dynamics enabling mRNA exchange, and Cajal bodies depend on scaffold protein interactions. This differential stability affects how each condensate type responds to membrane-mediated regulatory mechanisms. The formed condensates exhibit liquid-like properties, such as fusion, fission, and dynamic exchange of internal molecules, which can be quantitatively characterized using techniques like fluorescence recovery after photobleaching (FRAP) [9].

Recent studies have revealed broad and complex mutual regulation between lipid membrane organization and biomolecular condensates, reshaping cellular organization paradigms [10,11]. To deepen this understanding, we critically evaluate quantitative models of phase coupling, such as the Flory-Huggins theory adapted for membrane interfaces [12], which predict that interfacial tension reductions can lower nucleation barriers by up to 50%. This framework highlights underexplored variables like membrane curvature gradients, offering a constructive pathway for designing in silico simulations to test condensate stability under physiological perturbations. Thermodynamic phase coupling allows protein condensates to influence the stability of lipid domains, and conversely, these lipid domains aid in the nucleation and stabilization of condensate formation [6]. Additionally, membrane anchoring significantly reduces the critical concentration necessary for phase separation, from micromolar to nanomolar concentrations, thereby enhancing the spatial organization of cellular components [13].

Furthermore, the wetting of condensates on membrane surfaces, coupled with the resulting interfacial interactions, triggers membrane deformation processes that lead to diverse morphological changes, such as tubulation, budding, and interfacial ruffling spanning nano- to microscale scales [14]. The molecular basis of these phenomena is predicated on specific protein-lipid interactions. For example, when annexin A11 binds phosphoinositides, it induces lipid phase transitions. Concurrently, the wetting behavior of condensates on membranes is determined by the precise equilibrium between condensate-membrane affinity and interfacial tension [15]. Environmental factors, protein modifications, and cellular stress conditions serve as key regulatory elements, collectively modulating the strength and biological outcomes of these phase separation processes [16,17].

Post-translational modifications and cellular processes make membrane surfaces critical regulatory hubs for biomolecular phase separation [18]. For instance, phosphotyrosine-driven protein condensation can couple with membrane lipid phase transitions, creating organized signaling platforms through protein condensation [19]. Similarly, annexin A11 binds to phosphoinositides on lysosomal membranes, inducing lipid phase transitions that facilitate condensate-mediated membrane remodeling [15]. Three interconnected molecular mechanisms regulate lipid-condensate coupling. First, post-translational modifications, particularly phosphorylation, precisely control the critical concentration for condensate formation, phase separation propensity, and membrane affinity. For instance, phosphoserine-driven protein condensation in T-cell signaling pathways induces coupled lipid phase separation, demonstrating protein-lipid crosstalk [18,19]. Second, membrane surfaces function as control centers that reduce the threshold concentration required for condensate formation while simultaneously organizing lipid domains through thermodynamic coupling [6,18]. Third, environmental factors directly modulate these interactions. Calcium promote the phase separation of anionic phospholipids and alter membrane curvature through electrostatic effects [20]. Meanwhile, changes in membrane composition, including cholesterol content and acyl chain saturation, regulate lipid packing density, thereby controlling condensate-membrane affinity and wetting behavior [14,21,22].

These mechanisms have profound biological implication. Lipid-condensate coupling supports cellular signal transduction, organizes functional membrane domains, and enables dynamic responses to environmental stimuli [6]. However, imbalances in this regulation—such as lipid peroxidation, metabolic dysfunction, or pathological protein aggregation—can lead to aberrant lipid-condensate coupling [23]. These mechanistic insights drive therapeutic development targeting miomolecular condensates and their membrane interactions. Current strategies include cholesterol-modulating agents like statins, which alter membrane composition and lipid packing to disrupt pathological condensate-membrane interactions, as well as membrane-targeting compounds such as cyclodextrins that modulate lipid raft organization [14,24,25]. Advanced experimental technologies enable progress, including fluorescence correlation spectroscopy, super-resolution microscopy, in vitro reconstruction, and live-cell imaging, which provide detailed understanding of lipid-condensate interactions [21,26]. Despite progress, challenges persist, including limited quantitative descriptions of dynamics in complex cellular environments, absence of standardized protocols and metrics, and the gap between molecular mechanisms and clinical applications [24,27,28].

## 2. Fundamental Mechanisms of Lipid-Mediated Condensate Assembly

**Figure 1:**
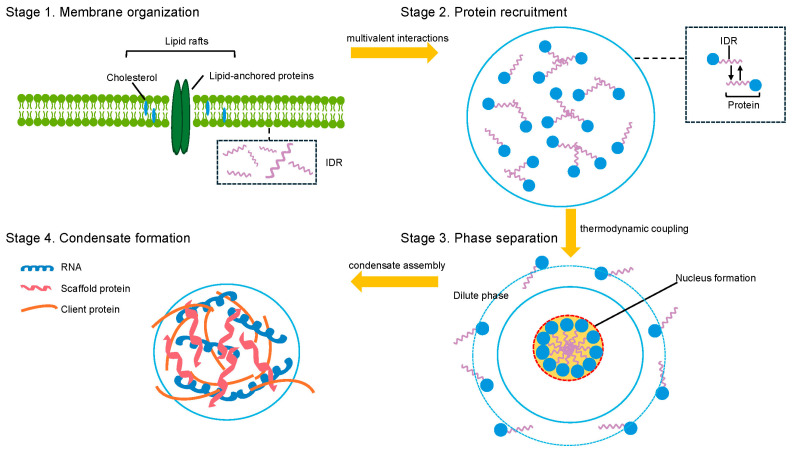
Mechanisms of lipid-mediated biomolecular condensate assembly. Green (lipid bilayer), blue (proteins), purple (IDR), orange (client protein), dark blue (RNA), red (scaffold protein).

The assembly of biomolecular condensates at membrane interfaces reflects an intricate interplay between protein phase separation and lipid organization, governed by distinct thermodynamic and kinetic principles that fundamentally differ from those of bulk solution condensation [29,30]. At the molecular level, membrane-associated condensate formation is driven by the synergistic integration of multivalent protein–protein interactions and specific protein-lipid binding events, establishing a unique physicochemical environment that significantly enhances phase separation propensity [6,18]. Lipid membranes serve as both a nucleation platform and a thermodynamic modifier, reducing the critical concentration for condensate formation by orders of magnitude—from typical micromolar levels in bulk solution to nanomolar concentrations at membrane surfaces [18]. This enhancement arises through multiple mechanisms: membrane confinement effects that elevate local protein concentrations, specific binding interactions between condensate-forming proteins and membrane lipids, and the cooperative stabilization of protein assemblies via membrane anchoring [31] (Figure 1).

The molecular architecture of membrane-associated condensates is fundamentally shaped by the amphiphilic nature of lipid interfaces, which establish distinct microenvironments characterized by unique electrostatic properties, hydration states, and molecular crowding conditions [32,33]. IDRs and LCDs of proteins exhibit enhanced phase separation propensity when proximal to membranes, driven by altered polymer-solvent interactions and the exclusion of water molecules at the lipid-protein interface [34,35]. Moreover, membrane curvature and lipid composition directly influence condensate assembly by modulating protein conformational dynamics and intermolecular interaction strengths [36,37]. Positively charged protein domains preferentially interact with anionic lipid headgroups, while hydrophobic regions embed into the membrane interface, forming multivalent anchoring points that stabilize condensate structures [38,39].

The thermodynamic coupling between protein phase separation and lipid domain formation constitutes a critical mechanism underlying membrane-mediated condensate assembly [6]. This coupling manifests through reciprocal stabilization: protein condensates can induce or enhance lipid phase separation by locally concentrating membrane-binding proteins, while lipid domains provide organized platforms that reduce the energetic barriers for protein condensation [40,41]. The strength of this coupling depends on specific molecular interactions between condensate components and membrane lipids, including electrostatic attractions, hydrogen bonding, and van der Waals forces [38]. Notably, the preferential partitioning of certain lipid species into condensate-associated membrane regions forms compositionally distinct domains with altered physical properties, such as modified membrane tension, curvature susceptibility, and protein binding affinity [42,43].

Dynamic regulation of membrane-mediated condensate assembly is achieved through multiple molecular switches responsive to cellular conditions and signaling events. Post-translational modifications, particularly phosphorylation and ubiquitination, significantly alter protein-membrane interactions by modifying electrostatic properties and binding specificities [44,45]. Environmental factors, such as pH, ionic strength, and metabolite concentrations, further modulate condensate assembly by influencing both protein–protein interactions and membrane physical properties [46]. Additionally, the presence of RNA molecules substantially enhances membrane-associated condensate formation by establishing multivalent protein-RNA networks that bridge membrane-bound and cytosolic components [47,48]. These regulatory mechanisms ensure precise control of membrane-mediated condensate assembly in response to cellular needs and environmental conditions, facilitating rapid and reversible transitions between assembled and disassembled states. For example, specific phosphorylation of the FUS low-complexity domain disrupts phase separation, aggregation, and toxicity, demonstrating how cellular modifications can prevent pathological condensate formation in neurodegenerative diseases like ALS [45].

## 3. Regulatory Mechanisms and Control Systems

**Figure 2:**
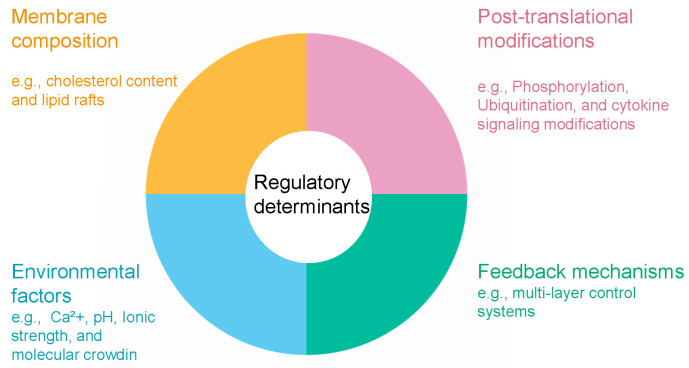
Regulatory determinants of lipid-condensate coupling.

The precise regulation of lipid-condensate coupling relies on complex multi-layered control systems that integrate molecular, cellular, and environmental signals to orchestrate dynamic responses in membrane organization and condensate behavior (Figure 2) [13,49]. At the post-translational level, phosphorylation serves as a primary regulatory mechanism, with kinase and phosphatase activities acting as reversible switches that control condensate formation, stability, and dissolution [50]. Phosphorylation events can promote or inhibit condensate assembly depending on the target residues and molecular context; for instance, phosphorylation of serine and threonine residues in intrinsically disordered regions typically suppresses condensate formation by introducing negative charges that disrupt multivalent interactions, whereas tyrosine phosphorylation enhances condensation by creating binding sites for SH2 domain-containing proteins [45,51]. This spatiotemporal control enables rapid modulation of condensate properties in response to signaling cascades, as exemplified by phosphotyrosine-driven LAT condensate assembly in T-cell receptor signaling [35].

Membrane composition acts as a fundamental control parameter governing condensate-membrane interactions through various biophysical mechanisms [14]. Cholesterol content directly modulates lipid packing density and membrane fluidity, thereby regulating the affinity and wetting behavior of protein condensates on membrane surfaces. This mechanism offers potential targets for lipidomics-based interventions to recalibrate membrane properties [52], thereby regulating the affinity and wetting behavior of protein condensates on membrane surface. Elevated cholesterol levels increase lipid chain order and decrease membrane permeability, forming more rigid domains that reduce condensate affinity due to diminished conformational flexibility of embedded proteins. In contrast, membranes with lower cholesterol or higher unsaturated fatty acid content exhibit enhanced condensate wetting and greater protein recruitment [52]. The asymmetric distribution of specific lipids across membrane leaflets adds further regulatory complexity, with phosphatidylserine and phosphoinositide in the inner leaflet serving as binding platforms for cationic protein domains [53].

Lipids contribute to condensate regulation beyond traditional bilayer configurations. Non-bilayer lipid phases, including hexagonal and cubic structures, interact with condensates to modulate their material properties and assembly dynamics [49]. These diverse lipid arrangements provide multiple mechanisms for fine-tuning condensate-membrane interactions in cellular environments.

Environmental control systems add further regulatory layers by modulating ionic strength, pH, and molecular crowding conditions [54]. Calcium ions play a crucial role by enhancing electrostatic interactions among anionic membrane lipids and inducing conformational changes in calcium-binding proteins that alter their condensation propensity [55,56]. Cellular pH variations profoundly affect the ionization states of protein residues and lipid headgroups, thereby tuning electrostatic interactions that drive condensate assembly [57]. Moreover, molecular crowding effects from high macromolecule concentrations amplify the thermodynamic driving force for phase separation while influencing membrane properties via osmotic stress [30,58]. For example, calcium, phosphorylation, and cholesterol collectively regulate CFTR protein cluster formation on cell membranes [55,56]. The integration of these diverse regulatory inputs occurs through complex feedback mechanisms, ensuring homeostatic control of condensate dynamics and enabling swift responses to cellular perturbations and stress [59].

## 4. Cellular Functions and Biological Significance

**Figure 3:**
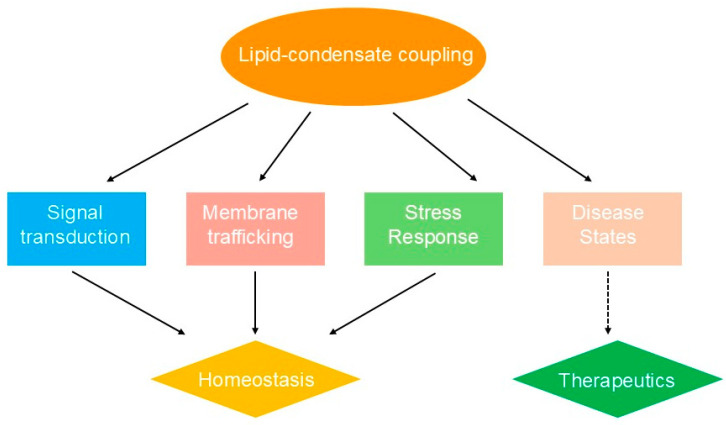
Lipid-condensate coupling in cellular processes and implications.

The coupling between lipid organization and biomolecular condensates plays critical roles in fundamental cellular processes, enabling advanced spatial and temporal control of biochemical reactions through the creation of specialized microenvironments [11]. In signal transduction pathways, membrane-associated condensates serve as dynamic signaling hubs that concentrate receptors, enzymes, and regulatory proteins to amplify and process signals [31,60]. The formation of signaling condensates at membrane surfaces generates locally enriched environments where substrate concentrations can exceed bulk cellular levels by several orders of magnitude, substantially increasing reaction rates and enabling ultrasensitive responses to stimuli [61]. Recent studies demonstrate that membrane-assisted condensates can enhance enzymatic rates by orders of magnitude through local concentration effects and molecular organization [61]. Coacervate droplets accelerate biochemical reactions by concentrating reactants and creating distinct physicochemical environments that influence reaction dynamics. This principle is illustrated in immune cell activation, where protein condensates at the immunological synapse organize receptor clustering and downstream signaling cascades while inducing coupled lipid phase separation to further enhance transduction efficiency [62] (Figure 3).

Membrane trafficking and organelle biogenesis represent additional cellular processes that critically rely on lipid-condensate coupling mechanisms [13]. The formation of specialized membrane domains via condensate-induced lipid phase separation facilitates the recruitment and organization of trafficking machinery, including coat proteins, motor proteins, and fusion apparatus [63,64]. Condensates can generate and stabilize membrane curvature by exerting localized forces and recruiting curvature-inducing proteins, thereby enabling vesicle formation and membrane remodeling [52]. Furthermore, the asymmetric distribution of condensate-forming proteins across membrane compartments contributes to organelle identity and function by establishing distinct biochemical environments that support specialized metabolic and regulatory processes [65,66].

The biological significance of lipid-condensate coupling extends to stress response and cellular adaptation mechanisms, where rapid reorganization of membrane domains and condensate assemblies allows cells to effectively respond to environmental challenges [67]. During cellular stress, the formation of stress granules and other protective condensates is often accompanied by alterations in membrane composition and organization that collectively promote cell survival [68,69]. Stress-induced changes in lipid composition can alter condensate properties and contribute to cellular dysfunction [69]. These coordinated responses involve the redistribution of essential cellular components into protective condensates while modifying membrane properties to preserve cellular integrity [70]. The reversible nature of these assemblies enables cells to swiftly return to normal function upon stress resolution, underscoring the adaptive advantages provided by lipid-condensate coupling systems [71].

Pathological disruption of lipid-condensate coupling mechanisms underlies numerous disease processes, highlighting the critical importance of these systems for cellular homeostasis [72]. Aberrant condensate formation in neurodegenerative diseases often involves dysregulated membrane interactions that promote pathological protein aggregation and membrane dysfunction [73,74]. For instance, the abnormal phase transition behavior of tau and TDP-43 proteins in intracellular condensates contributes to their pathogenic mechanisms of action in neurodegenerative diseases [73]. Similarly, metabolic disorders can perturb normal lipid composition and membrane organization, resulting in impaired condensate function and cellular abnormalities [58]. Insights into these pathological mechanisms have opened new avenues for therapeutic intervention by targeting condensate-membrane interactions, representing a promising frontier in precision medicine [24,75].

## 5. Experimental Approaches and Methodologies

The study of lipid-condensate coupling demands sophisticated experimental approaches capable of simultaneously probing membrane organization and condensate dynamics with high spatial and temporal resolution [11]. Fluorescence-based techniques form the cornerstone of current methodologies, with fluorescence recovery after photobleaching (FRAP) and fluorescence correlation spectroscopy (FCS) offering quantitative assessments of molecular dynamics within condensates and their exchange with surrounding environments [76,77]. Super-resolution microscopy methods, such as stimulated emission depletion (STED) microscopy and single-molecule localization microscopy (SMLM), have transformed the field by enabling visualization of condensate substructures and membrane domain organization at nanometer-scale resolution [78,79]. These techniques have uncovered previously hidden heterogeneities within condensates and demonstrated the presence of multiple condensate phases with distinct material properties [80].

Giant unilamellar vesicles (GUVs) and supported lipid bilayers serve as simplified membrane models, allowing systematic examination of how membrane composition, curvature, and surface charge influence condensate assembly [81,82]. In vitro reconstitution systems have become indispensable for dissecting the molecular mechanisms of condensate-membrane interactions under controlled conditions [9].

Microfluidic platforms provide precise manipulation of environmental parameters and real-time tracking of condensate formation kinetics, while also supporting high-throughput screening of potential modulators [83]. Advanced biophysical methods, including atomic force microscopy (AFM) and optical tweezers, yield direct measurements of condensate properties such as viscosity, surface tension, and mechanical stability [84]. Additionally, emerging techniques like holographic microscopy offer label-free alternatives for studying condensate dynamics, minimizing artifacts from fluorescent labeling [85]. For instance, surfactant-free PDMS-based microfluidic approaches have been developed to produce biomimetic GUVs with enhanced purity [81]. Optimized protocols for GUV production, stability assessment, and cargo loading have been established using microfluidic platforms [82]. Additionally, plug-and-play microfluidic systems utilizing droplet transfer across water-oil interfaces have enabled monodisperse GUV production [83].

Live-cell imaging approaches are crucial for corroborating in vitro observations and elucidating condensate behavior in physiological settings [86]. Advanced modalities, including light-sheet microscopy and lattice light-sheet microscopy, permit extended imaging of condensate dynamics with reduced phototoxicity, capturing formation, fusion, and dissolution events over prolonged periods [87,88]. Correlative light and electron microscopy (CLEM) supplies complementary ultrastructural details on condensate architecture and membrane organization [89]. Recently developed biosensors and optogenetic tools enable precise temporal manipulation of condensate assembly and disassembly, allowing exploration of their functional impacts in living cells [90,91]. The convergence of these diverse experimental strategies continues to deepen our understanding of condensate biology and uncover new therapeutic insights (Table 1).

## 6. Current Challenges and Future Directions

Despite significant advances in understanding lipid-condensate coupling, several fundamental challenges remain that limit both mechanistic insights and therapeutic applications [30]. Among these challenges, the lack of standardized characterization methods represents the most immediate barrier to progress, as it undermines the reliability of comparative studies and slows knowledge accumulation across the field. The intricate and dynamic cellular milieu presents substantial hurdles in quantitatively characterizing condensate attributes and their membrane interactions [46,92]. Current experimental approaches often rely on simplified model systems that may not fully recapitulate the complexity of cellular condensates, which typically contain hundreds of different proteins and RNA molecules with varying concentrations and interaction strengths [29]. The lack of standardized protocols and quantitative metrics for characterizing condensate properties across different experimental systems hampers reproducibility and comparative analysis between studies [93,94]. Furthermore, the transient and heterogeneous nature of many cellular condensates makes it challenging to establish clear structure-function relationships and predict the consequences of therapeutic interventions [95]. To address these experimental challenges, emerging approaches include: (1) development of standardized reference condensates with well-defined properties for method validation; (2) implementation of multi-modal imaging platforms that combine complementary techniques; and (3) establishment of quantitative frameworks for condensate property comparison across studies.

The translation of mechanistic understanding into clinical applications faces substantial obstacles related to target specificity and therapeutic delivery [96]. Many condensate-forming proteins are essential for normal cellular function, rendering the development of interventions that selectively perturb pathological condensates—while sparing physiological ones—challenging [97,98]. The tissue-specific and context-dependent nature of condensate function further complicates therapeutic targeting, as the same protein may participate in different condensates with distinct functions in various cell types [99]. Additionally, the delivery of therapeutic agents to specific subcellular compartments where condensates form remains a significant challenge, particularly for targeting membrane-associated condensates in specific organelles [35,100]. Potential solutions to these clinical translation challenges include: (1) development of condensate-specific biomarkers to distinguish pathological from physiological states; (2) design of tissue-targeted delivery systems using organ-specific promoters or nanocarriers; and (3) creation of conditionally active therapeutic agents that respond to disease-specific cellular environments.

Future research directions must address these challenges through the development of more sophisticated experimental tools and theoretical frameworks [101]. Advanced computational models that integrate molecular dynamics simulations with cellular-scale modeling will be essential for predicting condensate behavior in complex environments and designing targeted interventions [102,103]. Proposed Roadmap for Advancement: (1) Develop hybrid AI-molecular dynamics platforms to simulate lipid-condensate interactions at millisecond scales; (2) Establish international consortia for standardized condensate assays, including FRAP-based viscosity benchmarks; (3) Prioritize clinical trials of phase-modulating agents, such as annexin-targeted peptides, in phase-separated disease models like ALS. The development of new analytical techniques that can simultaneously probe multiple condensate properties in living cells will enable more comprehensive characterization of condensate function [104]. Furthermore, the establishment of standardized protocols and reference materials for condensate research will facilitate comparison between studies and accelerate progress in the field [105]. The integration of artificial intelligence and machine learning approaches holds promise for identifying novel therapeutic targets and predicting the effects of condensate modulation [106,107]. Advanced experimental methodologies, computational modeling, and clinical perspectives will be indispensable for harnessing the full therapeutic promise of lipid-condensate coupling mechanisms.

## 7. Therapeutic Implications

Lipid rafts and membrane domains serve as key platforms for condensate nucleation, making them attractive targets for pharmacological modulation. Statins reduce membrane cholesterol levels and lipid packing density, impairing aberrant condensate formation in amyloid-related diseases [24,25]. Cyclodextrins sequester cholesterol from membranes, disrupting lipid raft organization and reducing condensate wetting [14,24]. In neurodegenerative diseases, lipid peroxidation and raft dysregulation exacerbate pathological condensate formation [23,72]. Polyunsaturated fatty acids (PUFAs) increase membrane fluidity, reducing intrinsically disordered protein affinity for lipid domains, showing promise in metabolic disorders where altered lipid composition impairs insulin signaling condensates [22,58].

Small molecules targeting multivalent interactions can alter phase separation thresholds through disruption of π-π stacking or electrostatic forces [8]. High-throughput screening using microfluidic platforms has identified compounds targeting intrinsically disordered regions [83]. In cancer models, transcriptional condensate inhibitors disrupt super-enhancer interactions, suppressing oncogenic signaling [96,97,98,99]. Kinase inhibitors prevent phosphorylation-driven condensate formation, as demonstrated in T-cell signaling where phosphotyrosine motifs couple protein condensation to lipid phase transitions [18,19]. RNA-binding modulators can enhance or disrupt protein-RNA networks at membrane interfaces [47,48].

Effective therapeutics must overcome delivery and selectivity barriers. Condensates are often subcellular and transient, requiring targeted delivery systems such as nanoparticle-encapsulated agents that exploit membrane properties [49]. Off-target effects present challenges since many condensate proteins serve physiological roles [97]. Context-dependent condensate functions require personalized approaches, potentially guided by computational predictions [106,107].

Early clinical developments include condensate-targeting compounds in autoimmune disease models and membrane-anchoring inhibitors showing neuroprotective effects in neurodegeneration [73,74]. Future approaches may integrate condensate modulation with gene therapies, representing a paradigm shift in drug discovery for complex diseases.

## 8. Conclusions

The coupling between lipid membranes and biomolecular condensates represents a fundamental mechanism for cellular organization that enables precise control of biochemical processes and adaptive responses. Dysregulation of these interactions contributes to various diseases, including neurodegeneration and metabolic disorders. While therapeutic targeting of lipid-condensate coupling shows promise, challenges in selectivity and delivery remain. Future interdisciplinary approaches combining advanced experimental and computational methods will be essential for translating these mechanisms into clinical applications.

## Figures and Tables

**Table 1:** Methodological approaches for characterizing lipid-condensate interactions.

Technology Category	Specific Techniques	Primary Applications	Advantages	Limitations	Examples in Lipid-Condensate
Fluorescence-Based	FRAP, FCS	Measuring molecular dynamics and exchange rates	Quantitative, real-time data on diffusion and binding	Limited spatial resolution; requires labeling	Probing condensate fluidity and protein turnover in membrane-associated systems [76,77,84]
Super-Resolution Microscopy	STED, SMLM	Visualizing substructures and domains at nanoscale	High resolution (nanometers); reveals heterogeneities	Phototoxicity; complex data analysis	Imaging condensate phases and lipid domain organization [78,79,80]
In Vitro Reconstitution	GUVs, Supported Lipid Bilayers	Studying controlled interactions and assembly	Simplified models; tunable parameters (e.g., composition, curvature)	Lacks cellular complexity; potential artifacts	Examining effects of lipid charge on condensate wetting [14,81,82]
Microfluidic Platforms	Microfluidics with real-time monitoring	Kinetics analysis and high-throughput screening	Precise environmental control; scalable	Technical setup complexity	Screening modulators of condensate formation [83]
Biophysical Methods	AFM, Optical Tweezers	Measuring physical properties (viscosity, tension)	Direct mechanical insights; high precision	Invasive or low throughput	Assessing condensate stability and membrane deformation [52,84]
Label-Free Imaging	Holographic Microscopy	Dynamics without labels	Reduces labeling artifacts; non-invasive	Lower resolution than labeled methods	Studying condensate fusion without fluorescence interference [85]
Live-Cell Imaging	Light-Sheet Microscopy, Lattice Light-Sheet	Long-term dynamics observation	Minimal phototoxicity; 3D/4D imaging	Equipment cost; data volume	Tracking condensate assembly in signaling pathways [86,87,88]
Correlative and Advanced Tools	CLEM, Biosensors, Optogenetics	Ultrastructure and functional manipulation	Combines modalities; temporal control	Requires specialized expertise	Validating in vitro findings in immune cell activation [88,89,90]

## Data Availability

No new data were created or analyzed in this study. Data sharing is not applicable to this article.

## Abbreviations

The following abbreviations are used in this manuscript:

AFM	Atomic Force Microscopy
CLEM	Correlative Light and Electron Microscopy
FCS	Fluorescence Correlation Spectroscopy
FRAP	Fluorescence Recovery After Photobleaching
GUV	Giant Unilamellar Vesicle
IDR	Intrinsically Disordered Region
LCD	Low-Complexity Domain
LLPS	Liquid–Liquid Phase Separation
Lo	Liquid-Ordered
SMLM	Single-Molecule Localization Microscopy
STED	Stimulated Emission Depletion
